# Paracingulate sulcus morphology is associated with hallucinations in the human brain

**DOI:** 10.1038/ncomms9956

**Published:** 2015-11-17

**Authors:** Jane R. Garrison, Charles Fernyhough, Simon McCarthy-Jones, Mark Haggard, The Australian Schizophrenia Research Bank, Vaughan Carr, Ulrich Schall, Rodney Scott, Assen Jablensky, Bryan Mowry, Patricia Michie, Stanley Catts, Frans Henskens, Christos Pantelis, Carmel Loughland, Jon S. Simons

**Affiliations:** 1 Department of Psychology, University of Cambridge, Cambridge CB2 3EB, UK; 2 Behavioural and Clinical Neuroscience Institute, University of Cambridge, Cambridge CB2 3EB, UK; 3 Department of Psychology, Durham University, Durham DH1 3LE, UK; 4 Department of Cognitive Science, Macquarie University, Sydney, New South Wales 2109, Australia; 5 Department of Psychiatry, Trinity College, Dublin 2, Ireland; 6 University of New South Wales, Hospital Road, Randwick, New South Wales 2013; Australia; 7 University of Newcastle, University Drive, Callaghan, New South Wales 2308, Australia; 8 University of Western Australia, Stirling Highway, Crawley, Washington 6009, Australia; 9 Queensland Brain Institute, The University of Queensland, Brisbane, 4072 Queensland, Australia; 10 NeuRA, PO Box 1165, Randwick, New South Wales 2031, Australia; 11 Melbourne Neuropsychiatry Centre; 161 Barry St, Carlton, Vic 3053, Australia

## Abstract

Hallucinations are common in psychiatric disorders, and are also experienced by many individuals who are not mentally ill. Here, in 153 participants, we investigate brain structural markers that predict the occurrence of hallucinations by comparing patients with schizophrenia who have experienced hallucinations against patients who have not, matched on a number of demographic and clinical variables. Using both newly validated visual classification techniques and automated, data-driven methods, hallucinations were associated with specific brain morphology differences in the paracingulate sulcus, a fold in the medial prefrontal cortex, with a 1 cm reduction in sulcal length increasing the likelihood of hallucinations by 19.9%, regardless of the sensory modality in which they were experienced. The findings suggest a specific morphological basis for a pervasive feature of typical and atypical human experience.

## Abstract (web summary)

Hallucinations can occur in both healthy individuals and patients with psychiatric disorders. Garrison *et al*. here report that specific brain morphology differences in the paracingulate sulcus (PCS) can determine the occurrence of hallucinations in schizophrenia, irrespective of sensory modality.

## Graphical abstract

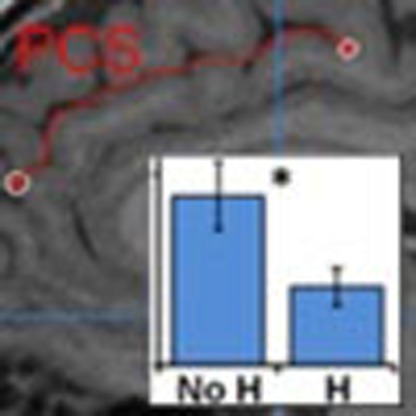

Hallucinations are a primary symptom of numerous mental health disorders, as well as featuring in the experience of many individuals within the healthy population. Previous attempts to characterize the brain mechanisms of hallucinations have often been confounded by designs relying on comparisons between patients and non-patients^1^. However, around 30% of patients who meet diagnostic criteria for schizophrenia never report such anomalous experiences, providing the potential for the discovery of brain structural markers that are specifically associated with the occurrence of hallucinations.

Undoubtedly, many neurobiological factors underlie hallucinations. In the present study, we focused on the paracingulate sulcus (PCS) in the medial prefrontal cortex (mPFC) given its previously established role in reality monitoring^2^, among other cognitive functions, defined as the ability to discriminate between real and imagined information^3^. Reality monitoring is impaired in patients with schizophrenia with hallucinations^4^,^5^,^6^ and in non-patients prone to hallucinations^7^. In the study by Buda *et al*.^2^, we previously identified that bilateral absence of the PCS was associated with reductions in reality monitoring performance in healthy individuals with no neurological damage. The PCS is one of the last sulci to develop *in utero*, providing the potential for individual differences in its morphology, such as sulcal length, to be particularly informative about functional variation in an area of the brain extensively implicated in reality monitoring judgments^8^,^9^.

Here, we investigate PCS length in both hemispheres in three matched groups: schizophrenia patients with a history of hallucinations, schizophrenia patients with no history of hallucinations and healthy controls (see Table 1 for participant details). PCS length was measured from structural MRI scans using a newly validated visual classification technique and data-driven whole-brain analysis methods, carried out blind to diagnosis (see Methods section for details). Examples of long and short PCS images are displayed in Fig. 1. We report converging results from across methods indicating that hallucination status can be determined by specific brain morphology differences in the PCS.

## Results

### Validation of PCS measurement protocol

To validate the new PCS measurement protocol, it was first applied to 53 healthy volunteer structural scans previously analysed by Buda *et al*.^2^, with the analysis undertaken blind to the ratings in the earlier study, to give 106 measurements of sulcal length across hemispheres. The left and right hemisphere PCS for each individual was classified as ‘prominent' if the length was >40 mm, ‘absent' if PCS length was <20 mm and ‘present' if PCS length fell between these two limits, based on the earlier protocols^2^,^10^. The PCS classifications obtained were then compared with the original ratings from the study by Buda *et al*.^2^ In all, 94 out of the 106 measurements matched precisely, resulting in a Cohen's Kappa of 0.79 (*P*<0.001), 95% CI (0.68, 0.84), indicating ‘substantial agreement'^11^ between the two protocols.

To validate the measurement protocol further, and verify its sensitivity to morphological variations in schizophrenia, we measured PCS length in a small, locally acquired independent sample of 19 patients with schizophrenia, all of whom experienced hallucinations, as well as in 19 matched control participants. Informed consent was obtained from these participants in a manner approved by the UK National Research Ethics Service. Total PCS length was significantly reduced in the patients with schizophrenia (mean=84.1 mm, s.d.=30.5 mm) compared with controls (mean=110.2 mm, s.d.=38.5 mm), *t*(36)=2.31, *P*=0.027, *d*=0.77. These independent validations provide grounds for confidence about the reliability of our measurement protocol, and the likelihood that it will be sufficiently sensitive to identify morphological differences in our larger sample of 153 patients with schizophrenia and controls that may distinguish those who experienced hallucinations from those without hallucinations.

### PCS measurement differences associated with hallucinations

Turning to the principal analysis of PCS morphology differences as a function of hallucination status, we compared PCS length between three large matched groups (patients with schizophrenia who had experienced hallucinations, patients with schizophrenia who had not experienced hallucinations and matched healthy controls; see Methods section for participant details and matching procedure). There was a main effect of group on total PCS length, summed across both hemispheres, *F*(2, 150)=8.90, *P*<0.001, *η*_*p*_^*2*^=0.106, which survived the addition of cortical surface area as a covariate, *F*(2, 149)=7.03, *P*=0.001, *η*_*p*_^*2*^=0.086. Other potential covariates such as age, IQ, intracranial volume and global brain gyrification index had no significant effect on PCS length and were removed from the model.

Planned comparisons revealed that patients with schizophrenia who experienced hallucinations exhibited significantly reduced PCS length compared with the patients without hallucinations (mean reduction=19.2 mm), *t*(111)=2.531, *P*=0.013, *d*=0.519 and healthy controls (mean reduction=29.2 mm), *t*(117)=4.149, *P*<0.001, *d*=0.805, whereas sulcal length between patients who did not experience hallucinations and healthy controls did not differ significantly, *t*(72)=1.07, *P*=0.288, *d*=0.246 (Fig. 2a).

With earlier research providing conflicting evidence of differential cortical-folding patterns between the two cerebral hemispheres in schizophrenia, we next investigated possible laterality effects on PCS length. There were main effects of hemisphere, *F*(1,150)=9.978, *P*=0.002, *η*_*p*_^*2*^=0.062, and group, *F*(2,150)=8.900, *P*<0.001, *η*_*p*_^*2*^=0.106, on PCS length, but no interaction between hemisphere and group. PCS length was greater in the left than the right hemisphere across all subject groups, *t*(152)=2.959, *P*=0.004, *d*=0.317 (Fig. 2b,c). Patients with schizophrenia who had experienced hallucinations exhibited reduced PCS length compared with the healthy controls in both hemispheres, *t*>2.636, *P*<0.01, *d*>0.546. The difference in PCS length between patients with schizophrenia who had experienced hallucinations and patients who had not experienced hallucinations was significant only in the left hemisphere, *t*(111)=2.464, *P*=0.015, *d*=0.505.

We tested the modality specificity of the observed relations by comparing PCS length between patients with auditory hallucinations and patients with hallucinations limited to other modalities (for example, visual, tactile, olfactory). The PCS reductions could not be differentiated according to hallucination modality, either summed across both hemispheres, *t*(77)=0.067, *P*=0.947, *d*=0.015, or within the left, *t*(77)=0.600, *P*=0.551, d=0.135, or right, *t*(77)=0.822, *P*=0.413, *d*=0.185, hemispheres alone, consistent with a generalized role for reality monitoring impairment in the formation of hallucinations, regardless of the sensory modality in which they occur.

Logistic regression was used to control for a number of potentially confounding demographic and clinical variables. Included in the analysis were left and right hemisphere PCS length, intracranial volume, cortical surface area, global gyrification index, time on antipsychotic medication, duration of illness and incidence of delusions. No variables aside from left hemisphere PCS length made a significant contribution to predicting participant group membership. A test of the revised model with non-significant terms eliminated against a constant-only model was statistically significant, indicating that left hemisphere PCS length reliably distinguished between hallucinating and non-hallucinating patients (*χ*^2^=5.848, *P*=0.016 with df=1), accurately categorizing the hallucination status of 70.8% of the patients with schizophrenia. The regression model predicts that, when left hemisphere PCS length is reduced by 10 mm, there is a 19.9% change in the odds ratio (95% CI=1.033 to 1.415), indicating the increased likelihood that patients with schizophrenia will experience hallucinations.

### Data-driven whole-brain analyses

To further validate the PCS measurement protocol and to determine whether between-group differences in PCS length were accompanied by structural variations elsewhere in the brain, we conducted separate automated whole-brain analyses of surface-based cortical gyrification and of voxel-based grey matter volume (see Methods section for details). Confirming the results of the PCS measurement method, significant differences in local gyrification index were observed in the mPFC regions of interest surrounding the PCS, namely bilateral frontopolar, medial orbitofrontal, superior frontal and paracentral cortices, with patients with schizophrenia who experienced hallucinations exhibiting significantly reduced gyrification in these regions compared with patients without hallucinations, *t*(111)=2.165, *P*=0.033, *d*=0.448 (Fig. 3). No significant regional group differences elsewhere in the brain survived correction for multiple comparisons.

Consistent with reductions in mPFC cortical folding in hallucinations, grey matter volume was significantly greater in the functionally defined 8-mm sphere mPFC region of interest surrounding the anterior PCS in patients with schizophrenia who experienced hallucinations than in those who did not (*x*=6, *y*=54, *z*=−5; BA 10; *Z*=2.82; *P*=0.036 (small volume corrected), Fig. 4). The region identified as significant using this voxel-based method was smaller than the region that emerged in the surface-based gyrification analysis, which may be attributable to the different properties of cortical morphology measured, as well as any of numerous statistical and methodological differences between the two techniques (see Methods section for details). In any event, no significant grey matter volume differences elsewhere in the brain, associated with the occurrence of hallucinations, survived correction for multiple comparisons.

## Discussion

Using newly validated visual classification techniques and automated, data-driven analysis methods, the present study identified that hallucinations were associated with specific brain morphology differences in the PCS region of the mPFC. Because the connection between PCS reduction and hallucinations was evident in participants who all had diagnoses of schizophrenia, our findings avoid confounding with patient status, as can occur in case–control comparisons. The hallucinating and non-hallucinating groups with schizophrenia in our study were matched for age, sex, handedness, IQ, duration of illness, antipsychotic medication and incidence of delusions and negative symptoms. In identifying that hallucinations can be distinguished by structural brain imaging data, we demonstrate that a multifactorial phenomenon which is defined experientially can be related to a single morphological change in the mPFC. As a tertiary sulcus forming around 36 weeks of gestation^12^, the 19.2 mm mean reduction in PCS length that distinguished patients who hallucinated from those who did not hallucinate might arise from genetic factors that influence primary folding of the cortex through a disruption to neurodevelopmental pathways. Alternatively, the variability in PCS length might be a non-genetic consequence of some disturbance in primary sulcal development, or might represent extremes of normal statistical variation in the development of primary and secondary sulci.

Our results go beyond previous findings of changes in cortical-folding patterns associated with schizophrenia. Several previous studies have reported differences in PCS morphology in patients with schizophrenia compared with healthy controls^13^,^14^, or investigated differences in global measures of cortical gyrification or sulcation associated with hallucination status^15^. The present study is the first to identify that PCS morphology changes can discriminate between hallucinating and non-hallucinating groups that are matched for overall brain volume, cortical surface area and global gyrification index, among other variables. The present findings are consistent with earlier research suggesting that leftward PCS hemispheric asymmetries in schizophrenia might be similar to those typically observed in healthy controls^14^,^16^, though some previous studies have reported reduced PCS asymmetry in schizophrenia^13^,^17^. In the present data, comparable laterality effects were observed in all subject groups, with significantly greater PCS length in the left than right hemisphere, and group differences evident across both hemispheres. Methodological differences might explain the discrepancies between previous studies, motivating the development of common measurement protocols, preferably incorporating both visual classification and automated, data-driven components, to optimize the identification and measurement of sometimes relatively indistinct or discontinuous anatomical landmarks such as the PCS.

Evidence from research in healthy individuals indicates that PCS reductions are associated with increased grey matter volume in the surrounding anterior cingulate cortex^18^, with Buda *et al*.^2^ reporting that increased grey matter volume in the mPFC correlated negatively with an individual's reality monitoring ability. Such findings fit with the present results, in which reduced mPFC surface-based gyrification and concomitant increased voxel-based grey matter volume were the only significant differences in the brain to be associated with the occurrence of hallucinations. Together with the results by Buda *et al*., these findings are consistent with a role for reality monitoring impairment in the generation of hallucinations, with a structural basis for that ability in the region of the PCS. An influence of reduced paracingulate folding and greater surrounding cortical volume may arise from weakened connectivity between the mPFC and both proximal and distal brain regions. Prominent theories of morphogenesis suggest that cortical folding in the human brain, which begins at around the 26th week of gestation^19^,^20^, results either from differential mechanical tension along white matter axons linking disparate brain areas^21^,^22^ or from variable tangential expansion of the cortical surface^23^.

Altered PCS morphology could thus lead to hallucinations through changes in connectivity between cortical regions involved in processing sensory representations and mPFC areas that support decision-making processes such as distinguishing real experiences from those that might have been imagined, among other cognitive functions^8^,^9^,^24^. This hypothesis has yet to be tested directly, although there is evidence of impaired anterior cingulate modulation of fronto–temporal connectivity in schizophrenia^25^. Investigating functional and structural connectivity between the broader mPFC and, for example, posterior auditory and language regions around the superior temporal gyrus, would further inform models of hallucination formation. Hallucinations are likely to be a multifactorial phenomenon^5^, and theoretical models implicate a range of cognitive and affective variables in their occurrence^26^,^27^. It is possible that modality-general risk factors, such as reduced PCS length, may interact in some individuals with modality-specific risk factors, such as reduced arcuate fasciculus integrity in the case of auditory hallucinations^28^, to produce hallucinations in specific sensory modalities. Information on neurodevelopmental models of schizophrenia could also be gained by comparing PCS morphology in family studies and during disease development.

Our findings support modality-general views of hallucinations as stemming from atypicalities in reality monitoring. They raise important questions for cognitive models of hallucinations including how the internal ‘raw material' of reality monitoring errors might be defined. In the case of auditory hallucinations, there is compelling evidence that hallucinations arise through the misattribution of internal events (for example, inner speech) as external auditory stimuli. A modality-general account would need to specify analogous internal events that could be misattributed as external ones in, for example, the visual or tactile modalities. A modality-general account would also have to explain considerable phenomenological variability in the experience as it is described in all modalities. Moreover, as with all theories proposing brain structural or functional changes associated with hallucinations, a reality monitoring account must explain why hallucinations are often transient phenomena rather than being experienced constantly. Susceptibility to hallucinations, and their triggering and maintenance by psychological and environmental factors, are likely to be multifactorial, complex processes. We show that a simple morphological variation is an important factor in determining why some individuals can have quasi-perceptual experience of entities that are not physically present.

## Methods

### Data set

T1-weighted MPRAGE structural MRI scans were obtained using a 1.5-T 3D GR/IR sequence (TR=1980, ms, TE=4.3 ms, flip angle=15°, FOV=256 mm × 256 mm × 176 mm, voxel size=1 mm^3^) from the Australian Schizophrenia Research Bank (ASRB) for 113 patients with schizophrenia and 40 matched healthy control individuals. All scans were of sufficient quality to enable the visual classification and automated, data-driven analyses to be undertaken. Participant demographic details and clinical information on which the groups were matched are presented in Table 1. Informed consent was obtained by the ASRB at recruitment, and the study was approved by the Macquarie University Faculty of Human Sciences Human Research Ethics Committee.

Patients with schizophrenia were allocated to groups based on their life-time experience of hallucinations as ascertained by direct clinical interview. Subjects in the first group reported experience of auditory hallucinations (*n*=39) or hallucinations in other modalities (*n*=40). Patients in the second group reported no life-time experience of hallucinations in any modality (*n*=34). The final group of subjects comprised healthy controls who were screened at registration for a family history of, or treatment for, psychiatric or neurological disorder (*n*=40). Patient groups were matched for IQ using the Wechsler Abbreviated Scale of Intelligence (WASI), and all groups were matched for age, sex and handedness. All analyses were carried out blind to participant group.

The original data set included one additional subject in the auditory hallucinations group but that individual's scan showed evidence of a neurological disorder and the subject was excluded from the analysis. A copy of the Diagnostic Interview for Psychosis^29^ and details of antipsychotic medication were also obtained for each subject, and key variables from these data are shown in Table 1. There were no significant differences between the patient groups in the duration of illness, duration of antipsychotic medication, in the types of delusions (out of a total of seven categories), or in the presence of negative symptoms (thought disorder or catatonia) that patients had experienced.

### Measurement of PCS length

**Figure 1:**
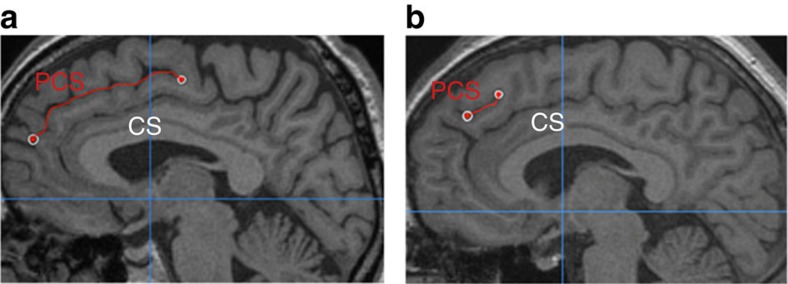
PCS measurement for two example images. The paracingulate sulcus (PCS), marked in red, lies dorsal and parallel to the cingulate sulcus (CS), itself dorsal to the corpus callosum. (**a**) In this image, the PCS is continuous and is measured from its origin in the first quadrant (indicated by the cross-hairs at *y*=0 and *z*=0) to its end. (**b**) In this example, the PCS appears less distinct; it is measured from the point at which it runs in a posterior direction, dorsal to the cingulate sulcus.

**Figure 2:**
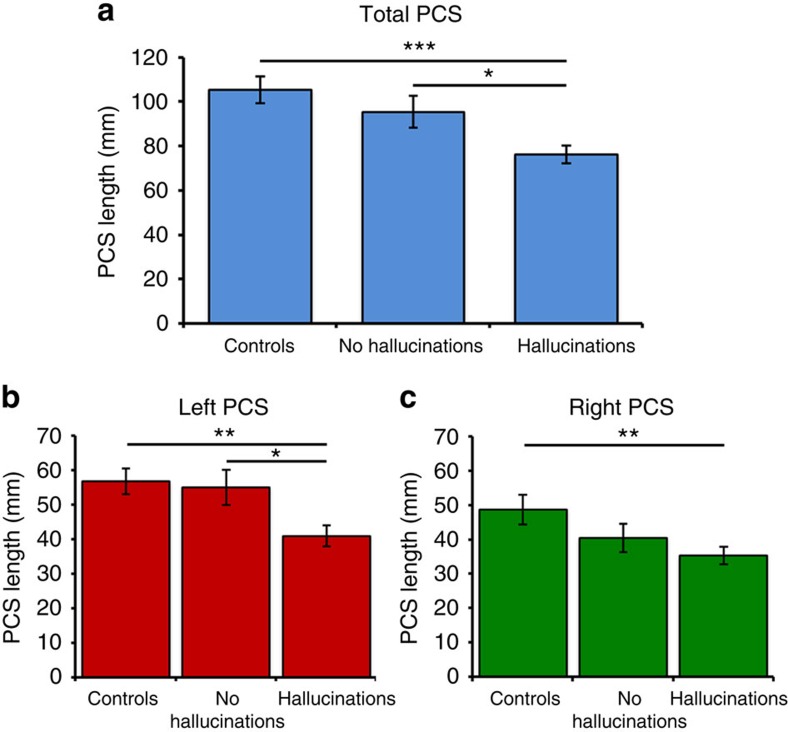
PCS length by group. (**a**) Total PCS length across both hemispheres. (**b**) PCS length in the left hemisphere. (**c**) PCS length in the right hemisphere. ***P<0.001, **P<0.01, *P<0.05. Error bars represent standard error of the mean. Controls: 40 healthy control subjects; no hallucinations: 34 patients with schizophrenia who had not experienced hallucinations; hallucinations: 79 patients with schizophrenia who experienced hallucinations in any modality.

**Figure 3:**
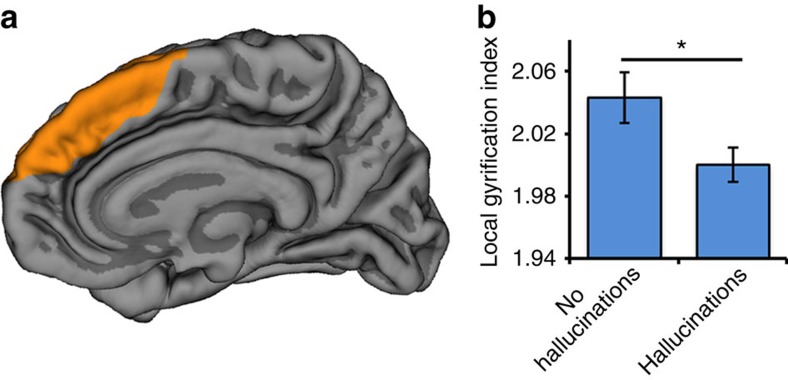
Whole-brain cortical gyrification differences as a function of hallucination status. (**a**) mPFC regions surrounding the PCS exhibiting significantly reduced gyrification in 79 patients who experienced hallucinations compared with 34 patients without hallucinations, rendered on a canonical pial cortical surface, viewed from the midline. (**b**) Local gyrification index in regions surrounding the PCS significantly differentiates patients with schizophrenia as a function of hallucination status, *t*(111)=2.165, *P*=0.033, *d*=0.448. Error bars represent standard error of the mean.

**Figure 4:**
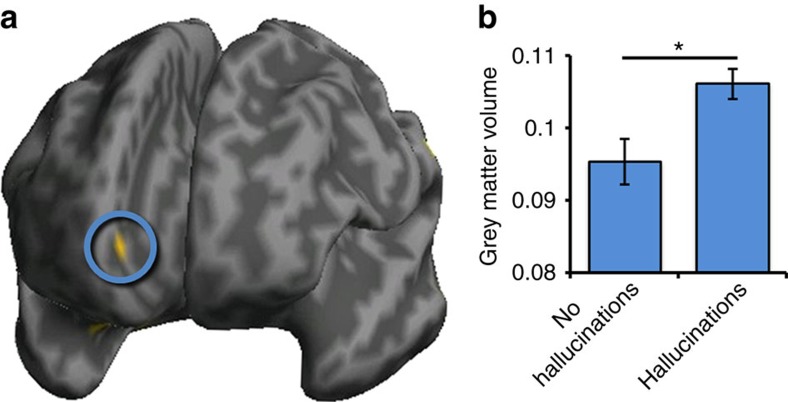
Grey matter volume differences measured with voxel-based morphometry. (**a**) Significantly greater grey matter volume in 79 patients who experienced hallucinations than in 34 patients without hallucinations in the mPFC region of interest in the vicinity of the anterior PCS (circled), rendered on an inflated canonical cortical surface, viewed from the front. (**b**) Grey matter volume in PCS region significantly differentiates patients with schizophrenia as a function of hallucination status, *Z*=2.82; *P*=0.036 (small volume corrected). Error bars represent standard error of the mean.

Individual scans were imported as DICOM folders into Mango brain visualization software (version 3.0; http://ric.uthscsa.edu/mango) and inspected for integrity. The PCS for each hemisphere was measured using the procedure described below, which was developed from that previously described^2^,^10^. In an axial view, the locations of the anterior and posterior commissures (AC and PC) were marked, and the scan rotated to line up the AC and PC in a horizontal plane. The scan was further translated as required to centre it, and the origin reset to the location of the AC. On a sagittal slice, 4 mm to the left or right of the medial line (*x*=±4), the cingulate sulcus (CS) was identified as the first major sulcus running in an anterior–posterior direction, dorsal to the corpus callosum and typically visible for five sagittal slices or more. Using a precise protocol, the PCS was then identified if present as a salient sulcus, running parallel, horizontal and dorsal to the CS, and visible for three or more sagittal slices measured from the medial (*x*=0) plane. The sulcus was measured using the ‘trace line' function in Mango from its origin in the first quadrant prescribed by *y*>0 and the horizontal line linking the AC and PC (*z*>0), starting at the point at which the sulcus ran in a posterior direction. The PCS was measured to its end point, which could fall outside of the first quadrant (see Fig. 1).

Where the PCS was discontinuous, a segment of sulcus was measured only if it started within the first quadrant, and additional segments were then included if total interruptions were <20 mm in length^10^. Any segments clearly running in a posterior–anterior direction were excluded but non-horizontal or parallel segments were included if the PCS was salient and running towards the exterior dorsal surface of the brain.

### Calculation of gyrification index

Measures of cortical folding across the brain were obtained by calculating the local gyrification index for each MRI structural scan using the method described by Schaer *et al*.^30^. The gyrification index gives a measure of cortical folding by comparing the amount of cortex buried within sulcal folds at the grey/white matter interface with the amount of visible or surface cortex at each vertex of the reconstructed brain surface, based on 3D spheres of radius 25 mm. Calculations made at each vertex can then be averaged to give a global gyrification index for each hemisphere, or for 34 individual brain regions as defined by an automated parcellation procedure^31^. Calculation was undertaken using FreeSurfer Software version 5.3.0 (http://surfer.nmr.mgh.harvard.edu/), which also provided measures of intracranial volume for each subject's scan. For statistical analysis, individual gyrification maps were registered to a template constructed by averaging the patient structural scans included in the study, and smoothed with a 5 mm full-width, half-maximum isotropic Gaussian kernel. Group differences in local gyrification were analysed by fitting a general linear model at each vertex on the surface. Age was included as a covariate, and non-parametric cluster-wise correction for multiple comparisons was performed using Monte Carlo simulation^32^, with a threshold of *P*<0.05, corrected for multiple comparisons across each hemisphere. Two-sample *t*-tests were used to identify region-specific differences averaged across *a priori* mPFC regions of interest in the vicinity of the PCS, the frontopolar, medial orbitofrontal, superior frontal and paracentral cortices^31^, at a threshold of *P*<0.05. Regional differences outside the region of interest were reported if they exceeded a threshold of *P*<0.05, corrected for multiple comparisons across the 34 parcellated brain regions.

### Voxel-based morphometry analysis

Whole-brain voxel-based grey matter volume differences were analysed using the VBM8 toolbox (http://dbm.neuro.uni-jena.de/vbm) implemented in SPM8 software (Wellcome Trust Centre for Neuroimaging, London, UK). Images were manually reoriented before tissue class segmentation and spatial normalization into Montreal Neurological Institute (MNI) stereotactic space^33^ were performed, using high-dimensional DARTEL image registration. The spatially normalized segmented images were modulated by the non-linear components derived from the normalization process, correcting for differences in total intracranial volume. Finally, images were spatially smoothed with an 8 mm full-width, half-maximum isotropic Gaussian kernel.

An *a priori* mPFC region of interest in the vicinity of the PCS was defined as an 8 mm sphere centred on coordinates reported in a recent fMRI study to be sensitive to reality monitoring manipulations in schizophrenia^9^. Two-sample *t*-tests using the General Linear Model in SPM8 were used to identify clusters in which grey matter volume differed between groups at a statistical threshold of *P*<0.05, corrected for voxels within the mPFC region of interest. Clusters outside the region of interest were reported if they exceeded a threshold of *P*<0.05, corrected for multiple comparisons across the entire brain.

## Additional information

**How to cite this article**: Garrison, J. R. *et al*. Paracingulate sulcus morphology is associated with hallucinations in the human brain. *Nat. Commun.* 6:8956 doi: 10.1038/ncomms9956 (2015).

## Figures and Tables

**Table 1:** Participant data.

	**Hallucinations**		**No hallucinations**	**Healthy controls**
	**Auditory**	**Other**				
*N*	39	40	34	40
Male (*N*)*	32	33	27	33
Female (*N*)*	7	7	7	7
Left handed (*N*)*	3	3	3	3
Age at scan (years)*	39.2 (10.6)	37.7 (8.9)	40.7 (9.8)	39.0 (10.3)
WASI-IQ†	100.7 (16.3)	101.7 (14.7)	105.6 (14.2)	119.6 (8.8)
Duration of illness (years)†	15.6 (9.0)	14.3 (7.6)	14.9 (10.3)	—
Medication (months)†	46.4 (38.1)	53.2 (31.3)	42.3 (35.8)	—
Delusion types†	2.6 (1.3)	2.9 (1.6)	2.8 (1.5)	—
Negative symptoms†	0.37 (0.6)	0.56 (0.6)	0.32 (0.6)	—

Note: data in parentheses denote s.d. Hallucinations: patients with schizophrenia who experienced hallucinations in the auditory modality (first column) or in other modalities (second column); no hallucinations: patients with schizophrenia who had not experienced hallucinations. Medication data were unavailable for four patients who experienced non-auditory hallucinations and two who had not experienced hallucinations. Delusion data were unavailable for one member of the latter group.

^*^Groups did not differ significantly in sex, handedness or age.

^†^Schizophrenia groups did not differ significantly in IQ, duration of illness, antipsychotic medication, or incidence of delusions or negative symptoms.
